# Improving coverage of civil registration and vital statistics, Bangladesh

**DOI:** 10.2471/BLT.18.219162

**Published:** 2019-07-23

**Authors:** Moyeen Uddin, Shah Ali Akbar Ashrafi, Abul Kalam Azad, Anir Chowdhury, Hafizur Rahman Chowdhury, Ian Douglas Riley, Andres Montes, Martin Bratschi, Carla AbouZahr, Zeaul Alam

**Affiliations:** a CRVS, Data for Health Initiative, Cabinet Division, Room #1212, Level #11, Govt. Transport Pool Bhaban, Secretariat Link Road, Dhaka-1000, Dhaka, Bangladesh.; b Directorate General of Health Services, Dhaka, Bangladesh.; c Access to Information a2i Program, ICT Division, Dhaka, Bangladesh.; d Global Burden of Disease Group, University of Melbourne, Melbourne, Australia.; e Public Health Programs, Vital Strategies, New York, United States of America.; f Bloomberg Data for Health Initiative, Geneva, Switzerland.; g Coordination and Reforms, Cabinet Division, Dhaka, Bangladesh.

## Abstract

**Problem:**

Bangladesh has no national system for registering deaths and determining their causes. As a result, policy-makers lack reliable and complete data to inform public health decisions.

**Approach:**

In 2016, the government of Bangladesh introduced a pilot project to strengthen the civil registration and vital statistics system and generate cause of death data in Kaliganj Upazila. Community-based health workers were trained to notify births and deaths to the civil registrar, and to conduct verbal autopsy interviews with family members of a deceased person. International experts in cause-of-death certification and coding trained master trainers on how to complete the international medical certificate of cause of death. These trainers then trained physicians and coders.

**Local setting:**

Kaliganj Upazila has an estimated population of 304 600, and 5600 births and 1550 deaths annually. Health assistants and family welfare assistants make regular visits to households to track certain health outcomes.

**Relevant changes:**

Following the start of the project in 2016, the number of births registered within 45 days rose from 873 to 4630 in 2018. The number of deaths registered within 45 days increased from 458 to 1404. During this period, health assistants conducted 7837 verbal autopsy interviews. Between January 2017 and December 2018, 105 master trainers and more than 7000 physicians were trained to complete the international medical certificate of cause of death and they completed more than 12 000 certificates.

**Lessons learnt:**

Training community-based health workers, physicians and coders were successful approaches to improve death registration completeness and availability of cause-of-death data.

## Résumé

**Problème:**

Le Bangladesh n'est doté d'aucun système national pour enregistrer les décès et déterminer leurs causes. Les responsables politiques ne disposent donc pas de données fiables et complètes pouvant servir de base à la prise de décisions en matière de santé publique.

**Approche:**

En 2016, le gouvernement du Bangladesh a mis en place un projet pilote destiné à renforcer le système des registres et des statistiques de l'état civil et à obtenir des données sur les causes de décès dans l'upazila de Kaliganj. Des agents sanitaires de proximité ont appris à notifier les naissances et les décès à l'officier de l'état civil, et à réaliser des autopsies verbales auprès des membres de la famille d'une personne décédée. Des experts internationaux de la certification des causes de décès et du codage ont appris à des formateurs principaux à remplir le certificat médical international indiquant la cause du décès. Ces formateurs ont ensuite formé des médecins et des codeurs.

**Environnement local:**

L'upazila de Kaliganj compte quelque 304 600 habitants et enregistre environ 5600 naissances et 1550 décès par an. Des aides-soignants et des auxiliaires en matière de protection de la famille effectuent des visites à domicile régulières pour suivre certains résultats cliniques.

**Changements significatifs:**

À la suite du lancement du projet en 2016, le nombre de naissances enregistrées en 45 jours est passé de 873 à 4630 en 2018. Le nombre de décès enregistrés en 45 jours est passé de 458 à 1404. Au cours de cette période, les aides-soignants ont réalisé 7837 autopsies verbales. Entre janvier 2017 et décembre 2018, 105 formateurs principaux et plus de 7000 médecins ont appris à remplir le certificat médical international indiquant la cause du décès, et ils ont rempli plus de 12 000 certificats.

**Leçons tirées:**

La formation d'agents sanitaires de proximité, de médecins et de codeurs s'est révélée efficace pour améliorer l'enregistrement des décès et accroître la disponibilité de données sur les causes de décès.

## Resumen

**Situación:**

Bangladesh no tiene un sistema nacional para registrar las muertes y determinar sus causas. Como resultado, los responsables de formular políticas carecen de datos fiables y completos que sirvan de base para la toma de decisiones en materia de salud pública.

**Enfoque:**

En 2016, el gobierno de Bangladesh introdujo un proyecto piloto para fortalecer el registro civil y el sistema de estadísticas vitales y generar datos sobre las causas de muerte en Kaliganj Upazila. Se capacitó a los trabajadores sanitarios de la comunidad para que notificaran los nacimientos y las defunciones al registro civil, y para que realizaran entrevistas de autopsia verbal con los familiares de una persona fallecida. Expertos internacionales en la certificación de la causa de muerte y la codificación de formadores expertos capacitados sobre cómo completar el certificado médico internacional de causa de muerte. Estos formadores luego capacitaron a médicos y codificadores.

**Marco regional:**

Kaliganj Upazila tiene una población estimada de 304 600 habitantes y 5 600 nacimientos y 1 550 defunciones anuales. Los asistentes sanitarios y los asistentes de bienestar familiar realizan visitas periódicas a los hogares para hacer un seguimiento de ciertos resultados sanitarios.

**Cambios importantes:**

Tras el inicio del proyecto en 2016, el número de nacimientos registrados en un plazo de 45 días aumentó de 873 a 4 630 en 2018. El número de muertes registradas en 45 días aumentó de 458 a 1 404. Durante este periodo, los asistentes sanitarios realizaron 7 837 entrevistas de autopsia verbal. Entre enero de 2017 y diciembre de 2018, 105 formadores expertos y más de 7 000 médicos recibieron capacitación para completar el certificado médico internacional de causa de defunción y completaron más de 12 000 certificados.

**Lecciones aprendidas:**

La capacitación de los trabajadores sanitarios, los médicos y los codificadores de la comunidad fueron enfoques exitosos para mejorar la integridad del registro de defunciones y la disponibilidad de datos sobre la causa de la muerte.

## ملخص

**المشكلة:**

ليس لدى بنغلاديش نظام وطني لتسجيل الوفيات وتحديد أسبابها. لذلك يفتقر واضعو السياسات إلى بيانات موثوقة وكافية لإبلاغ قرارات الصحة العامة.

**الأسلوب:**

قدمت حكومة بنغلاديش في عام 2016 مشروعًا رياديًا لتعزيز نظام السجل المدني والإحصاءات الحيوية وإيجاد بيانات عن أسباب الوفاة في كاليغانغ أوبزيلا. تم تدريب العاملين الصحيين في المجتمع على إبلاغ المواليد والوفيات للسجل المدني، وإجراء مقابلات التشريح اللفظي مع أفراد عائلة الشخص المتوفى. قام خبراء دوليون في إصدار شهادات أسباب الوفاة وعمليات الترميز بتدريب كبار المدربين على كيفية إكمال الشهادة الطبية الدولية لسبب الوفاة. ثم قام هؤلاء المدربون بتدريب الأطباء والمبرمجين.

**المواقع المحلية:**

يبلغ عدد سكان كاليغانغ أوبزيلا 304600 نسمة، بمعدل 5600 حالة ولادة، و1550 حالة وفاة سنويًا. يقوم المساعدون الصحيون ومساعدو رعاية الأسرة بزيارات دورية للعائلات لتتبع نتائج صحية معينة.

**التغيّرات ذات الصلة:**

بعد بدء المشروع في عام 2016، ارتفع عدد المواليد المسجلين في غضون 45 يومًا من 873 إلى 4630 حالة في عام 2018. وارتفع عدد الوفيات المسجلة في غضون 45 يومًا من 458 إلى 1404 حالة. أجرى المساعدون الصحيون في هذه الفترة 7837 مقابلة تشريح لفظي. في الفترة ما بين يناير/كانون ثاني 2017 وديسمبر/كانون اول 2018، تم تدريب 105 من كبار المدربين وأكثر من 7000 طبيب على إكمال الشهادة الطبية الدولية لسبب الوفاة، وقد أكملوا بالفعل أكثر من 12000 شهادة.

**الدروس المستفادة:**

كان تدريب العاملين الصحيين في المجتمع والأطباء والمبرمجين طريقة ناجحة لتحسين استكمال تسجيل الوفيات وتوافر بيانات سبب الوفاة.

## 摘要

**问题:**

孟加拉国没有登记死亡和确定死亡原因的国家系统。因此，政策制订者缺乏可靠、完整的数据来为公共卫生决策提供信息。

**方法 2016:**

年，孟加拉国政府推出了一个试点项目，旨在强化民事登记和人口动态统计系统，并生成 Kaliganj Upazila 地区的死因数据。社区卫生工作者接受培训，向民事登记员通报出生和死亡情况，并与死者家属就尸检结果进行口头会谈。死因确认和编码领域的国际专家就如何完成国际死亡证明书死因诊断对主要培训师进行了培训。随后再由这些培训师培训医生和编码员。

**当地状况:**

Kaliganj Upazila 约有 304 600 人，每年出生约 5600 人，死亡约 1550 人。健康助理和家庭福利助理定期走访家庭，跟踪某些健康结果。

**相关变化 自:**

2016 年项目启动后，45 天内登记的出生人数从 873 人增加到了 2018 年的 4630 人。45 天内登记的死亡人数从 458 人增加到了 1404 人。在此期间，健康助理进行了 7837 次尸检口头会谈。2017 年 1 月至 2018 年 12 月期间，105 名主要培训师和 7000 多名医生接受了如何完成国际死亡证明书死因诊断方面的培训，并完成了 12000 多份证明。

**经验教训:**

培训社区卫生工作者、医生和编码员是提高死亡登记完整性和死因数据可用性的成功方法。

## Резюме

**Проблема:**

В Бангладеш отсутствует национальная система регистрации смертей и определения их причин. В результате политическое руководство не имеет надежных и полных данных для принятия информированных решений в области здравоохранения.

**Подход:**

В 2016 году правительство Бангладеш внедрило пилотный проект, направленный на усовершенствование системы регистрации актов гражданского состояния и статистики естественного движения населения и создание базы данных о причинах смертности в подокруге Калигандж. Местных медицинских работников научили сообщать о фактах смерти и рождения в отдел регистрации актов гражданского состояния, а также обучили проведению вербальной аутопсии в форме опроса членов семьи умершего лица. Международные специалисты в области сертификации причин смерти и кодирования обучили основных инструкторов тому, как правильно заполнять медицинское свидетельство о причине смерти. Эти инструкторы затем обучали врачей и кодировщиков.

**Местные условия:**

В подокруге Калигандж проживает около 304 600 человек, ежегодный показатель случаев рождения составляет 5600, а смерти — 1550. Помощники санитарного врача и помощники специалистов по вопросам бытового обслуживания регулярно посещают семьи и отслеживают определенные вопросы, связанные с состоянием здоровья.

**Осуществленные перемены:**

После запуска проекта в 2016 году количество фактов рождения, регистрируемых в течение 45 дней, выросло с 873 до 4630 в 2018 году. Количество фактов смерти, регистрируемых в течение 45 дней, выросло с 458 до 1404. За этот период помощники санитарного врача провели 7837 вербальных аутопсий в форме опросов. За период с января 2017 года по декабрь 2018 года 105 инструкторов и более 7000 врачей прошли обучение правильному заполнению международного медицинского свидетельства о причине смерти и заполнили более 12 000 таких свидетельств.

**Выводы:**

Обучение местных медицинских работников, врачей и кодировщиков оказалось успешным подходом к улучшению полноты регистрации фактов смерти и доступности данных о причинах смерти.

## Introduction

In 2018, Bangladesh had a population of approximately 165 million, of these around 104 million (63%) people were living in rural areas and about 48 million (29%) people were younger than 14 years of age.^1^ Since gaining independence in 1971, the country has achieved significant reductions in child and maternal mortality and life expectancy has increased from an estimated average of 46 years to over 70 years in 2015.^2^ However, the government needs reliable and up-to-date information on natality and mortality to guide policy and planning at the national and subnational levels. The most efficient way of generating such data is a national civil registration system, which officially registers all births and deaths on a continuous basis and generates vital statistics.

In 2004, a new birth and death registration Act was promulgated and in 2010, an online birth and death registration was introduced.^3^ However, by 2013 only 39 646 births had been registered, representing 1.3% of the expected 3 million births. Between the inception of the online registration system in October 2010 and February 2014, the cumulative total deaths registered amounted to 103 443, compared with the expected 761 000 deaths in 2013 alone.^4^

An assessment of the civil registration and vital statistics system in 2013 identified multiple reasons for the low registration rates of deaths and births, including absence of mechanisms for reporting home births and deaths; inadequate understanding among health facility staff of the need to report births and deaths; lack of awareness in the population of the value of registration; cumbersome registration procedures; and overlap and inconsistencies between paper-based and electronic registration.^5^

The country’s seventh Five-Year Plan 2016–2020 calls for a stronger civil registration and a vital statistics system to produce timely and complete birth and death data for the entire country.^6^ Here we describe a pilot project to increase birth and death registration and generate reliable information on causes of death.

## Setting

In 2016, the government estimated that some 15% of all deaths take place in health facilities and 85% in homes, the latter with limited or no medical supervision.^7^ Although deaths in public-sector health facilities are reported through the health management information system, these deaths are not notified to the civil registrar and therefore not officially registered. Moreover, the causes of most of these deaths are not determined according to international standards as defined by the World Health Organization (WHO).

The government introduced a pilot project to strengthen the civil registration and vital statistics system and generate cause of death data in Kaliganj Upazila. This administrative area has an estimated population of 304 600 (2011 census), and approximately 5600 births and 1550 deaths annually.^8^

## Approach

In 2016, the government established a national civil registration and vital statistics coordinating group to oversee efforts to increase birth and death registration. The group consisted of stakeholders in health, civil registration, statistics, local government, information and technology, justice, legal affairs and the Cabinet Division, which is responsible for interministerial coordination. With support from the Bloomberg Data for Health Initiative, the group introduced several priority activities: (i) technical support for strengthening the office of the registrar general; (ii) training of health assistants and family welfare assistants to increase notification and registration of births and deaths in the pilot area; (iii) implementation of verbal autopsy to ascertain causes of deaths in the pilot area; (iv) utilization of the international form of the death certificate and medical certification of cause of death in hospitals across the country; and (iv) training and capacity development for statistical coding and analysis of mortality statistics.

In the pilot area, local government, health and relevant administrative officials and community representatives discussed the importance of birth and death registration and agreed to the intervention in the area. The health ministry designated health assistants and family welfare assistants as intermediaries between families and the civil registrar. These assistants make regular visits to households in their areas of operation to track the health of women and children (including pregnancy outcomes, immunization and child growth and development). Assistant health inspectors trained the assistants to record births and deaths during their household visits and to notify the events to the local registration office for official registration within 45 days of occurrence. The assistants helped the family to complete the registration forms and counselled them where to collect the associated certificates. They also counselled families about the importance of visiting the registration office to complete the registration and collect the relevant certificates.

**Fig. 1:**
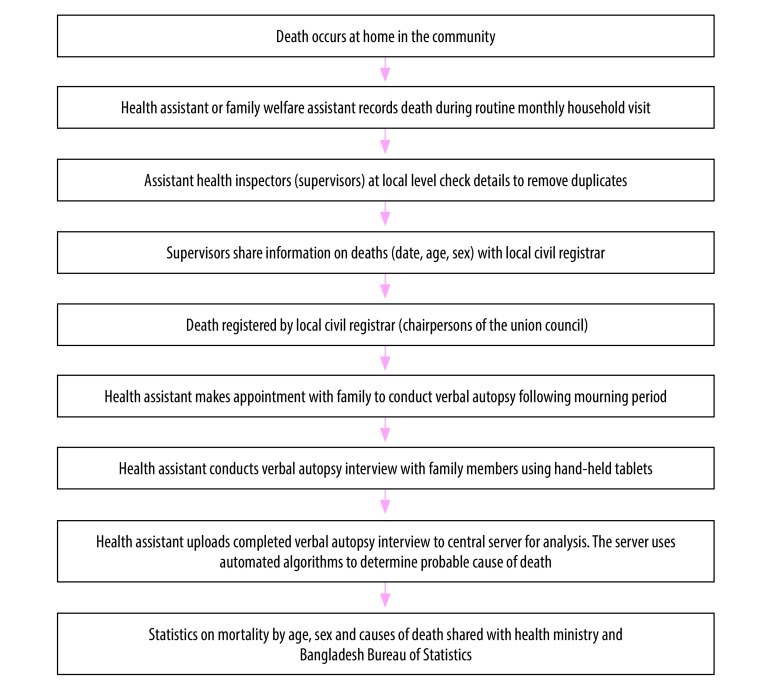
Death notification and verbal autopsy implementation processes, Bangladesh, 2016

In addition, when a death was identified, the health assistants arranged to meet with the family after the mourning period to conduct a verbal autopsy interview (Fig. 1). Small cash payments were provided to the health assistants and to their supervisors to compensate for the additional transport costs.

The health assistant used SmartVA, a tool developed by the institute for Health Metrics and Evaluation at the University of Washington. The tool consists of a verbal autopsy questionnaire and the Tariff2 automated diagnostic algorithm to identify probable cause of death. The health assistants uploaded completed interviews to a central server that ran the diagnostic algorithms and shared the probable cause of death with the health management information system. On ethical and confidentiality grounds, and in agreement with the communities involved, individual cause of death information from the verbal autopsy was not shared with either the family members or with the health assistants.

To complement the cause of death statistics from the verbal autopsy, the health ministry introduced WHO’s International Form of Medical Certification of Cause of Death (2016 version).^9^ The ministry used a cascade training approach, by first creating a cadre of master trainers to be responsible for the training of physicians nationwide. Starting in January 2017, experts in cause of death certification and coding from the University of Melbourne and the regional WHO Collaborating Centre in Sri Lanka conducted workshops for master trainers using training materials based on international standards adapted to local circumstances. Master trainers, who were physicians in 11 hospitals in urban and rural areas, learnt to complete the medical certificate of cause of death in accordance with international standards. At the same time, the health ministry established a new cadre of health information officers to carry out statistical coding of the underlying cause of death in accordance with the *International statistical classification of diseases and related health problems, 10th revision* (ICD).^10^ Since ICD statistical coding is a new area of work in Bangladesh, the coding is being introduced through WHO’s *Start-up Mortality List*.^11^

## Relevant changes

In the pilot area, the timeliness birth and death notification and registration have improved. In 2016, a total of 873 births were registered within 45 days, corresponding to an estimated completeness level of 16%. Following the intervention, the numbers of births registered within 45 days rose to 3401 in 2017 and 4630 in 2018. By the end of 2018, birth registration completeness had reached 83%.

The number of deaths registered within 45 days in Kaliganj was 458 in 2016, corresponding to an estimated completeness level of 30%. Following the intervention, deaths registered increased to 1339 in 2017 and 1404 in 2018, with death registration completeness reaching 91% in 2018.

By the end of 2018, health assistants had conducted 7837 verbal autopsy interviews on deaths of 7424 adults, 239 children and 174 neonates.

Between January 2017 and December 2018, 105 master trainers and over 7000 physicians have been trained to complete the international medical certificate of cause of death. The physicians have completed more than 12 000 certificates and the data have been entered into the district health information system database.^12^ The health ministry is now gradually rolling out medical certification of cause of death to all public and private hospitals. Analysis of the newly available cause of death data is currently under way.

## Lessons learnt

Here we show that the Kaliganj model is an effective and efficient strategy for increasing birth and death notification and registration, as well as for generating cause-of-death data in settings with little or no data on cause of death patterns (Box 1). Health assistants and family welfare assistants rapidly became adept at using electronic devices to collect verbal autopsy interviews and this enabled the production of cause-of-death data within two to three months of death registration. This success suggests that this model is a feasible strategy for long-term, large-scale mortality surveillance and can be integrated within the civil registration system.^13^ Replication of the interventions in additional administrative areas could significantly improve national birth and death registration completeness.

Box 1 **Summary of main lessons learnt**

• Community-based health workers can, with appropriate training and support, successfully identify births and deaths, and notify them to the local civil registrar for official registration.

• Community-based health workers can conduct verbal autopsy interviews with family members using hand-held devices and upload the data to a central server that generates plausible cause-of-death distributions.

• In health facilities, physicians can be trained to correctly complete the international form of the medical certificate of cause of death.

The government has invested financial and in-kind domestic resources to extend the pilot approach to a purposive sample of all subdistricts of Gazipur district and one sub-district of Mymensingh district (Trishal), covering almost 2 million people. In this expanded area, a total of 660 verbal autopsy interviewers and supervisors had been trained by the end of 2018 and over 18 453 verbal autopsies had been conducted. Analysis of cause of death distributions for the registered deaths is ongoing. Discussions are under way regarding the most efficient and sustainable sampling strategy for taking verbal autopsy implementation to scale nationwide.^14^

The government has positioned civil registration and vital statistics as central to its goal of enhancing service delivery across the life course, simplifying administration and strengthening statistical systems. The use of health assistants and family welfare assistants as notification agents has led to significant increases in birth and death registration and generated data on deaths by age, sex and causes in settings where most deaths occur at home without medical supervision. Complementing these data with information on causes of deaths that occur in hospitals will provide empirical data on the evolution of the epidemiological transition in Bangladesh. 

The Kaliganj model calls for multiple interventions across many sectors and levels, a high degree of interministerial collaboration, resources, technical knowledge and information, and technology support. A key finding from the pilot phase is the importance of sustained, high-level political leadership and commitment across sectors, particularly between health, the office of the registrar general and the Bangladesh Bureau of Statistics. The cabinet division has been pivotal by taking on a major role in policy advocacy, stakeholder coordination and progress monitoring.
